# Training emotional competencies at the workplace: a systematic review and metaanalysis

**DOI:** 10.1186/s40359-024-02198-3

**Published:** 2024-12-04

**Authors:** Miriam Mehler, Elisabeth Balint, Maria Gralla, Tim Pößnecker, Michael Gast, Michael Hölzer, Markus Kösters, Harald Gündel

**Affiliations:** 1 Department of Psychosomatic Medicine and Psychotherapy, Ulm University Hospital, Ulm, Germany; 2 Center for Burnout and Stress-Related Disorders, Privatklinik Meiringen, Meiringen, Switzerland; 3 Sonnenbergklinik, Stuttgart, Germany; 4 Center for Evidence-Based Healthcare, Medical Faculty Carl Gustav Carus, Technical University Dresden, Dresden, Germany

**Keywords:** Emotional intelligence, Empathy, Emotion regulation, Workplace intervention, Meta-analysis, Leadership, Training and development

## Abstract

Recent systematic reviews have shown that emotional competencies can be improved through training. In the workplace, such training has become increasingly popular over the last decade. These programs aim to enhance emotional intelligence, empathy or emotion regulation. This study wants to assess the training effects and potential moderators of these workplace interventions. To our knowledge, this is the first systematic review that focuses on the workplace context and integrates emotional intelligence, empathy, and emotion regulation training interventions. This study has been preregistered with PROSPERO and a protocol has been published before the review was conducted (CRD42021267073). We conducted a systematic literature search using Embase, PsycInfo, PSYNDEX, Web of Science and the Cochrane Central Register of Controlled Trials. The included studies were analyzed in two metaanalyses. In the primary analysis, we analyzed standardized mean changes in emotional competencies before and after the training for 50 included studies, depending on (a) training construct and (b) participants’ profession (teachers, health professionals, managers, and others). To determine the efficacy of the trainings, we conducted a separate metaanalysis of controlled trials only (*k* = 27). Both metaanalyses yielded moderate overall effect sizes that also persisted more than three months after the training end: (1) *SMD*_pre-post_ = 0.44 (95% *CI* [0.29, 0.59]), (2) *SMD*_EG-CG_ = 0.46 (95% *CI* [0.30, 0.63]). All professions benefited equally from the interventions and we observed no significant differences in the effectiveness of emotional intelligence, empathy, and emotion regulation trainings. Overall, our results suggest that workplace interventions effectively train emotional competencies, regardless of profession or specific training focus. Limitations are the high heterogeneity and the low methodological quality of the studies analyzed. Our study shows the need for more high-quality studies, like randomized controlled trials. Additionally, companies may consider incorporating emotional competence training into their employee and leadership development programs routinely. This study was preregistered on PROSPERO (CRD42021267073).

**Supplementary Information:**

The online version contains supplementary material available at 10.1186/s40359-024-02198-3.

## Introduction

Emotions and relationships play a crucial role in life and personal well-being, including in the workplace. Emotional Intelligence (EI) is a key ability needed to manage stress, motivate team members, and foster a trusting, cooperative team environment [1]. Relationships, on the other hand, are an influential protective factor regarding mental and physical health [2, 3]. Recurring conflicts, a loss of motivation, or increasing sick days might be consequences when emotions and relationship issues are ignored over a long time [4–6]. De Dreu and Gelfand [7] stressed out that factors such as increasing pressure to change, globalization of economies, remote work, and increased interdependency within work teams contribute to heightened conflict potential and uncertainty in the workplace. Therefore, soft skills like EI are nowadays of particular importance for individuals, teams, as well as organizations.

### Importance of emotional competencies

The importance of emotional competencies is also widely supported by research, with several meta-analyses and systematic reviews highlighting how higher levels of EI are associated with improved physical and psychological health [8, 9], better relationship quality [10, 11] and different work-related outcomes like constructive conflict management [12, 13], less occupational stress [13], better job performance [14–16], authentic leadership [17], higher job satisfaction [18], more organizational commitment and fewer turnover intentions of their employees [19]. Higher levels of empathy are also related to enhanced personal relationships and prosocial behavior [20]. The team managers’ empathy or EI skills are associated with employees well-being, satisfaction and motivation [19, 21] as well as their employees ratings of their performance [22, 23]. Functional emotion regulation strategies, like cognitive reappraisal or expressive suppression, are furthermore associated with mental health [24] and with team innovation when used by team leaders [25]. In sum, research demonstrates the importance of emotion-related abilities and skills also in the context of work.

### Theoretical approaches to emotional competencies

Various emotion-related constructs contribute to our understanding of emotional functioning, particularly in workplace contexts. EI, empathy, and emotion regulation are among the most prominent in both research and training, with EI receiving significant attention over the past decade. EI is typically defined as the ability to perceive, understand, manage, and use emotions [26]. However, three different theoretical approaches to EI exist: the ability, trait, and mixed models. Each model has practical implications for the training design and the evaluation of training effectiveness. The ability model describes EI as an actual ability related to cognitive intelligence. Ability EI is typically assessed using tests of maximal performance, such as the Mayer-Salovey-Caruso Emotional Intelligence Test (MSCEIT-2.0) [27]. In contrast, the trait model views EI as part of one’s personality, representing self-perceived emotional abilities [28]. Tools like the Trait Emotional Intelligence Questionnaire (TEIQue) measure these traits, often referred to as emotional self-efficacy [29]. Finally, mixed models define EI as a broader, learned capability, often also named emotional competence [30, 31]. These models incorporate both skills, personality traits, motivation and affect and link EI to specific competencies in the workplace, such as self-awareness and empathy [32]. Because mixed models also include non-emotional factors, like motivation, its definition of emotional competencies slightly differs from a traditional understanding. Common tools to assess mixed EI are the Emotional Competence Inventory (ECI) [33] or the Emotional Quotient Inventory (EQ-I; 28). Unlike ability EI, both trait and mixed EI assessments typically rely on self-report or peer-report measures. While the terms “EI” and “emotional competencies” are often used interchangeably, EI refers more specific to the cognitive potential to develop skills across the four emotional competencies clusters, whereas emotional competencies are more behaviorally oriented and focus on the practical application of emotional skills in daily life.

Empathy is another crucial socio-emotional construct with a multidimensional nature and varied definitions [34]. It is often described as the ability to understand and share another person’s feelings while recognizing them as distinct from one’s own [34, 35]. Davis’s Interpersonal Reactivity Index (IRI; 32) captures this complexity through two cognitive (Perspective Talking and Fantasy) and two affective dimensions (Empathic Concern and Personal Distress). Another widely used measure is the Jefferson Scale of Empathy [36].which focuses on the relevance of empathy in clinical and educational settings. Like EI, empathy has also been critiqued for concerns regarding its construct validity [34]. While some mixed EI models include empathy as one among many subcomponents, this approach may overlook its complex, multidimensional nature. Therefore, defining and measuring empathy as a distinct ability allows for a more targeted and comprehensive evaluation.

Finally, the concept of emotion regulation focuses on the conscious and unconscious processes by which individuals influence their own emotions [37]. Gross’s process-oriented model of emotion regulation focuses on the strategies employed for the self-regulation of emotions and is often measured with tools like the Emotion Regulation Skills Questionnare (ERQS) [38]. This conceptualization of emotion regulation differs from the emotion management facet of ability EI. While Gross’ model focuses exclusively on self-regulation, EI researchers examine emotion both within oneself and in interactions with others. Additionally, the emotion regulation dimensions within EI tend to be more outcome-oriented, emphasizing the effectiveness of emotional management strategies [39]. Although EI and emotion regulation are distinct theoretical constructs, they are interrelated: research indicates that different levels of EI are associated with varying patterns of emotion regulation strategies [40]. For instance, individuals with high EI are more likely to confront rather than avoid negative situations and are generally more effective at regulating their emotions in interpersonal contexts, in contrast to individuals with lower EI levels [40, 41].

This overview of the various emotion-related constructs highlights that clear conceptual delineation is sometimes challenging. The constructs of EI, emotional competencies, but also empathy, are often criticized for their broadness and issues with discriminant validity. Self-report instruments assessing typical performance, such as the EQ-i or SREIS, tend to correlate strongly with each other, as do performance tests, like the MSCEIT or STEM [42–46]. However,, correlations between these two types of instruments are much weaker [42, 43, 46]. This raises the question about whether maximum-performance tools like the MSCEIT and typical-performance measures like the SREIS—though both linked to the ability model of EI—truly capture the same ability. Some reseachers have therefore suggested a classification based on assessment type: maximum versus typical performance [43]. Additionally, some studies also indicate intercorrelations among self-report measures of empathy, emotion regulation, and EI, which complicates efforts to define and differentiate these constructs conceptually [47–50].

Nevertheless, the concepts outlined above form the foundation for many workplace interventions. Building on the outlined research, it is important to examine how these distinct yet overlapping constructs are applied in training contexts. This allows us to determine whether the different theoretical foundations lead to unique training approaches and varying levels of effectiveness, or if, in practice, they converge on similar content and techniques. For clarity, we will use"emotional competencies" as an umbrella term throughout this paper to refer to the different emotional abilities – EI, empathy and emotion regulation.

### Emotional competencies trainings

The recent popularity of EI and other emotion-related soft skills has led to a variety of workplace interventions [14]. Interventions exist in several contexts, such as clinical, educational or organizational, and with training focuses on different emotional skills. The metaanalysis of Hodzic and colleagues [51] included 28 EI interventions with treatment- and control group. They found moderate overall effect sizes of *SMD*_pre-post_ = 0.51 and concluded that EI can be trained. Mattingly and Kraiger [52] metaanalyzed 50 pre-post-interventions training EI in adults. The effect size across all samples was *SMD*_pre-post_ = 0.61. These results support previous findings [53, 54]. Both studies included working, non-working and student samples. Van Berkhout and Malouff metaanalyzed 18 randomized controlled trials of empathy training, with no restrictions on sample type. Their findings suggest that empathy trainings are also effective with moderate effect sizes (*g* = 0.63). Another metaanalysis on teaching clinical empathy to medical students also exists, showing moderate positive training effects [55]. Regarding emotion regulation training, we found no systematic reviews or meta-analyses focusing specifically on non-clinical adult samples. However, some interventions in school settings suggest that emotion regulation skills can be effectively taught to both adults and children [56].

All the mentioned metaanalyses included participants from various backgrounds, especially student samples. To our knowledge, this is the first metaanalysis specifically concentrating on emotional competency trainings within the workplace setting, and thus offering insights tailored to the demands and dynamics of professional environment. Employees and students differ in key ways: Employees generally have greater age, life, and work experience—factors that might influence training effectiveness. Research shows that age moderates training effects and is positively associated with emotional competencies such as empathy, emotion regulation and emotional intelligence [57–62]. Several studies showed that the motivation to learn and transfer knowledge was higher for older participants [62, 63], although neural plasticity declines in the ageing brain and thus reduces the brain’s ability to learn [64].

Furthermore, workplace interventions often target unique needs like leadership, conflict management, and team dynamics, which are less applicable to student populations and may result in different effectiveness across settings. The workplace also offers frequent exposure to emotionally challenging situations, providing employees with more opportunities to practice emotional skills. Most likely emotion-laden life experiences moderate the association between age and emotional competencies [65, 66]. Thus, employees may enter training with higher levels of these competencies than students and may also have more opportunities to apply what they learn. Also, professions with high interpersonal demands, like those in healthcare, may require greater emotional skills, leading to potentially greater training benefits compared to roles with focus on task oriented or technical responsibilities [20]. In light of this background, researchers expressed the need for further systematic investigations to explore the factors that determine who can benefit from EC trainings [51, 56]. Our metaanalysis aims to address this question and to further investigate if training effects vary depending on the profession. Insights from this study can guide HR departments and policymakers in choosing the most effective training approaches and developing professional development initiatives that specifically address the emotional demands of various roles.

*Research question 1: Are controlled workplace interventions effective in training emotional competences?*

*Research question 2: Do the effect sizes differ according to the profession of the training participants?*

Research findings suggest that enhancements in EI may not be immediately evident following training interventions but may manifest over time [67, 68]. Emotional competencies seem relatively stable and need some time to undergo detectable change [69]. Exploring the long-term effects of EC training allows us to evaluate observable changes in EC over time and examine the persistence of training effects. This investigation is crucial for practitioners and organizations seeking to optimize the design and implementation of EC trainings in the context of employee development.

#### Research question 3: Do training effects persist across time?

As described above, there are many different interventions training global EI, facets of EI, empathy, or emotion regulation that are based on different theoretical models. Given the overlap among these distinct constructs, it remains unclear to what extent the methods and content of the various training programs differ or resemble each other. Burke and Day [70] demonstrated that training methods influence training outcomes. So far, no empirical research exists in order to compare the described concepts and their training effects. Our metaanalysis aims to extend previous research that focused on only one of these concepts. By bringing together these closely related constructs, we seek to deepen our understanding and explore potential similarities as well as the differences in training methods and effectiveness. Examining how those emotion-related constructs are applied in the workplace can help organizations make better-informed decisions about training focus, objectives and methods used, distinguishing between specific competencies.

*Research question 4: Do training contents and effect sizes differ depending on the emotion construct the training is based on?*

In sum, this metaanalysis aims to address these questions providing a comprehensive examination of workplace interventions targeting emotional competencies, including not only EI but also empathy and emotion regulation. This analysis seeks to provide a nuanced understanding of the effectiveness of emotional competency training in the workplace. Additionally, by focusing specifically on interventions conducted within organizational settings, this study aims to offer practical insights that can inform both the research and development of evidence-based training programs for enhancing emotional competencies among employees and leaders. Studies, as mentioned above, have shown that emotional competencies are highly significant in the workplace. However, for companies, it is crucial to determine whether offering such training programs actually results in improvements in the targeted skills—essentially, evaluating the return on investment of these training efforts. Moreover, we want to provide recommendations for future intervention research by emphasizing methodological considerations and identifying new avenues for exploration. Our study may also offer insights for organizations on common, effective training methods in workplace settings and suggest whether these approaches should be customized based on employees' specific professions.

## Methods

This metaanalysis has been preregistered with PROSPERO and a protocol has been published before the review was conducted (CRD42021267073). We report this metaanalysis following the Preferred Reporting Items for Systematic Reviews and Meta-Analyses (PRISMA) guidelines [71].

### Eligibility criteria

We included all prospective interventions that evaluated the training effects of workplace interventions aiming at the improvement of different facets of emotional competencies with at least one data collection point before and after the intervention, or through comparison of post-test-scores with a control group. We had no restrictions regarding the year of dissemination or language.

According to our research interest, only interventions with employed participants older than 18 years old were included. Consequently, we excluded interventions conducted in academic settings. The mixed model of EI describes different facets belonging to the broader concept of emotional competencies [30]. In our search strategy, we aimed to include interventions focused on training EI, emotion regulation, empathy, but also conflict management and emotional awareness. In general, the objective of included interventions needed to be the improvement of at least one facet of emotional competencies. We specified that they should include at least one measure specifically designed to assess emotional competencies. Consequently, conflict management trainings should be also included when the ability to manage conflicts constructively was described as emotional skill, training contents also contained information regarding emotion management and at least one measure assessed emotional competencies. The measure of emotional competence had to be validated. To confirm reliability and internal validity, we examined each study's methods section and cross-referenced relevant validation research (e.g. [43]). Furthermore, reports of means and standard deviations of the emotional competences scores or enough statistical information (e.g., *t*- or *p*-values) to estimate effect sizes were necessary. More detailed information regarding our PICO-criteria can be found in Table A in the Appendix.

Since we did not identify any studies on emotional awareness and conflict management trainings that met our inclusion criteria, contrary to our expectations, we have decided not to include a detailed discussion of these topics in the introduction and subsequent sections of the paper.

### Literature search

A sensitive search strategy was developed based on subject headings (MeSH) and keywords (see Appendix Table B). We selected both psychological and multidisciplinary databases to ensure a comprehensive search. We identified relevant studies using the electronic databases EMBASE, PsycInfo, PSYNDEX, Web of Science and the Cochrane Central Register of Controlled Trials (Central). Reports of trials were also obtained from international trial registers via the World Health Organization’s trials portal (The International Clinical Trials Registry Platform) and via ClinicalTrials.gov. Furthermore, we screened related reviews for relevant studies. PROSPERO was checked for ongoing or recently completed systematic reviews and metaanalysis. If there were relevant publications, references were checked for additional cited articles. The initial search was conducted in June 2021 and the last update was done in November 2022.

### Study selection process

In the first step of the study selection, titles and abstracts of the literature were screened by two independent researchers against the inclusion criteria. Studies that met the inclusion criteria were rated as potentially relevant and the full texts were obtained. If full reports weren’t available, we contacted the study authors via mail. The two reviewers then screened the full texts and assessed the eligibility of the studies. If necessary, information was missing, we contacted the study authors. Reasons for exclusion of reports were recorded. Disagreements between reviewers were resolved through discussion.

### Data collection process

We developed a coding scheme based on the recommendations of von Elm [72]. Detailed descriptions of the extracted variables and the data used for the analyses are provided in the Appendix (Tables C & D). The scheme contains items to describe study design, training-, and participants’ characteristics. Besides control variables for the evaluation of the study quality, data to calculate pre-post- as well as comparative effect sizes for our defined outcomes and moderators were extracted. Data extraction was done by one researcher and subsequently checked by another.

### Analyses

We defined the pre-post effect size of emotional competencies within four weeks after the end of the intervention as the primary endpoint. As the time to the first assessment after training was longer in many of the trials, we decided to perform further analyses for all time periods. If there were different outcome measures available, preference was given to endpoint data from the ability-model of EI. As both EI and empathy are conceptualized as multidimensional constructs, different facets are often assessed. To ensure statistical independence, we refrained from using multiple effect sizes from a single study. Analyzing each dimension separately could inflate the sample size, so we opted to aggregate a total score of emotional competencies where possible. When studies provided subscale scores, we averaged the effect sizes of these subscales to derive an overall effect size for the study, recognizing that this method may obscure specific improvements in certain facets. For all included measures, a higher score implies stronger emotional competencies.

Pre-post effect sizes were calculated as standardized mean difference (SMD), standardized by the group pretest standard deviation [73]. Pre-post correlations, which are necessary to calculate the variance of pre-post effect sizes, are rarely reported and therefore had to be estimated. Contrary to our considerations of the study protocol, we decided to calculate our effect size with an assumed correlation of *r* = 0.7. This decision is based on the results of studies I n the field of EI that found a correlation of 0.56–0.76 between pre- and post-measurement [67, 74].

Subgroup analyses for the construct and profession of the participants were calculated. The variable “profession” consisted of the groups “teachers”, “health professionals” (e.g. nurses, physicians), “managers” and “others”. The groups “emotional intelligence”, “empathy” and “emotion regulation” belong to the variable “construct”.

To determine the efficacy of the interventions, we also conducted an additional analysis based on controlled trials only. Effect sizes were calculated as Hedges’*g* for comparative effects.

Effect sizes for both pre-post- and comparative comparisons were weighted by the inverse variance and aggregated in random-effects models. Study heterogeneity was assessed by the *I*^2^-parameter and tested by Chi^2^-tests. Due to high heterogeneity, we conducted additional outlier and influence diagnostics to identify and address potential sources of bias or inconsistency in the data [75]. Cook’s distance was used to identify outliers which were subsequently excluded in sensitivity analysis. Publication bias was graphically analyzed by funnel plots and statistically by Egger’s regression test. For data analyses we used RevMan [76] and the metafor package [77] in R Studio [78].

In order to assess the risk of bias within the studies included we used the Risk of Bias In Non-randomized Studies of Interventions-I (ROBINS-I) tool [79]. Studies were rated as *low, moderate*, *serious* or *critical* risk of bias in the following bias domains: (a) confounding, (b) selection of the participants, (b) classification of interventions, (c) deviations from intended interventions, (d) missing data, (e) measurement of outcomes and (f) selection of the reported results. A study was judged to be at overall low risk of bias if all domains were at low risk. The rating of serious risk of bias resulted when the study was judged to be at serious risk of bias in at least one domain, but not at critical risk of bias in any domain. This assessment was made by two separate reviewers. Disagreement was solved by discussion. Robvis visualization tool was used for the visualization of the results [80].

## Results

### Study selection

**Fig. 1:**
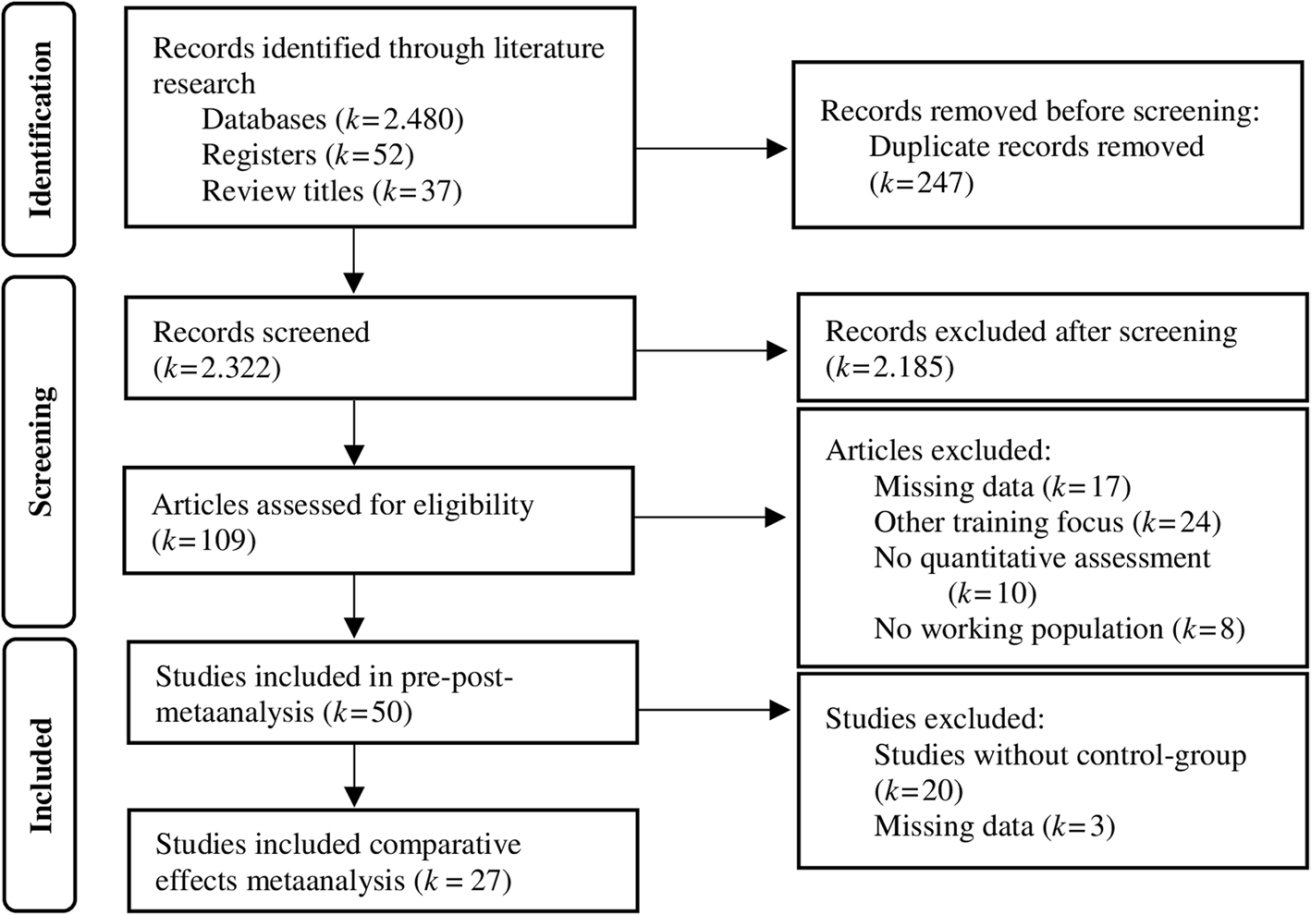
PRISMA Flowchart demonstrating the study selection process

We identified a total of 2569 studies. After title and abstract screening, full texts were obtained. We retrieved and screened 109 reports. Of these, 58 articles were excluded because (a) of missing data for the calculation of effect sizes (e.g. [81, 82], (b) the intervention did not focus on the training of emotional competences [83], (c) the population also consisted of people without work (e.g. [69, 84, 85] (d) the assessment of emotional competencies was not quantitative or with unvalidated measures [82, 86]. Finally, 50 studies met the inclusion criteria and were included in pre-post-metaanalysis. The metaanalysis of comparative effects included 27 eligible studies with a control group. The flow diagram of Fig. 1 illustrates the selection process.

### Sample, study and intervention characteristics

The pre-post-metaanalysis included *k* = 50 samples with an overall sample size of *N* = 3233. For *k* = 41 the time after training end until the first follow-up assessment was within four weeks. Table 1 shows the distribution of main study and training characteristics of all 50 included studies. You can find detailed study and training characteristics, as well as the data used for the analyses for each included study [87–126], in Table D of the Appendix.

**Table 1:** Study and training characteristics of the 50 studies included in pre-post-metaanalysis

Study characteristics		
Publication type	Peer-reviewed journals Dissertations Books Conference paper^1^	*82% (k* = *41)* *12% (k* = *6)* *2% (k* = *1)* *4% (k* = *2)*
Country	Europe United States Australia Eurasia South America Africa	*27% (k* = *19)* *31% (k* = *16)* *14% (k* = *7)* *12% (k* = *6)* *2% (k* = *1)* *2% (k* = *1)*
Study design	Control group Pre-post RCT	*48% (k* = *24)* *40% (k* = *20)* *12% (k* = *6)*
Follow up	No follow-up With follow-up 1 / 2 / 3 follow-ups	*74% (k* = *37)* *26% (k* = *13)* *k* = *8 / 3 / 1*
Measures	Self-report Peer-report / 360—report Ability test	*68% (k* = *34)* *18% (k* = *9)* *14% (k* = *7)*
EI Model	Mixed Model Ability Model Trait Model	*70% (k* = *26)* *24% (k* = *9)* *6% (k* = *2)*
Training characteristics		
Training duration	Mean days of training Mean hours of training	*5.4 (from 1 to 30)* *19*
Training period	Mean duration in days	*96 (from 1 day to 2 years)*
Methods		
Lectures	No lectures / not specified Lectures in person Online lectures	*14% (k* = *7)* *84% (k* = *42)* *2% (k* = *1)*
Coaching	No coaching / not specified Personal coaching session Coaching through team member	*82% (k* = *42)* *16% (k* = *8)* *2% (k* = *1)*
Feedback	No feedback / not specified Feedback by group Feedback by trainer Feedback by test results	*54% (k* = *27)* *16% (k* = *8)* *12% (k* = *6)* *26% (k* = *13)*
Group discussions	No discussions / not specified Group discussions	*46% (k* = *23)* *54% (k* = *27)*
Homework	No homework / not specified Practices Diary E-learning platform	*62% (k* = *31)* *26% (k* = *13)* *12% (k* = *6)* *4% (k* = *2)*
Practices	No practices / not specified EI strategies Role play Mindfulness techniques Art-based methods Adventure-based methods	*24% (k* = *12)* *58% (k* = *29)* *32% (k* = *16)* *14% (k* = *7)* *6% (k* = *3)* *2% (k* = *1)*

^1^A conference paper is a scholarly presentation created by a researcher or expert for an academic or professional conference, documenting the research, findings, or ideas

Most of the included trainings focused on EI (*k* = 41), followed by empathy interventions (*k* = 7). Only two interventions trained emotion regulation. There were no interventions training emotional awareness or conflict management that met our inclusion criteria. There were several applied measures. For EI interventions the EQ-I (*k* = 12) and the MSCEIT (*k* = 4) were used most often, for empathy interventions the Jefferson Scale of Empathy (*k* = 4) or the Interpersonal Reactivity Index (*k* = 3) and for emotion regulation interventions the Emotion Regulation Skills Questionnaire (*k* = 2). Besides emotional competencies, General Well Being (GHQ-12, Goldberg & Williams, 2011), perceived stress (PSQ; Levenstein et al., 1993), burnout (Maslach Burnout Inventory, Maslach, Jackson & Leiter, 1997) or job satisfaction were assessed in several studies.

The focus of implemented training methods was on cognitive-verbal techniques. But there were also ten trainings that integrated non-verbal methods.

### Risk of bias

**Fig. 2:**
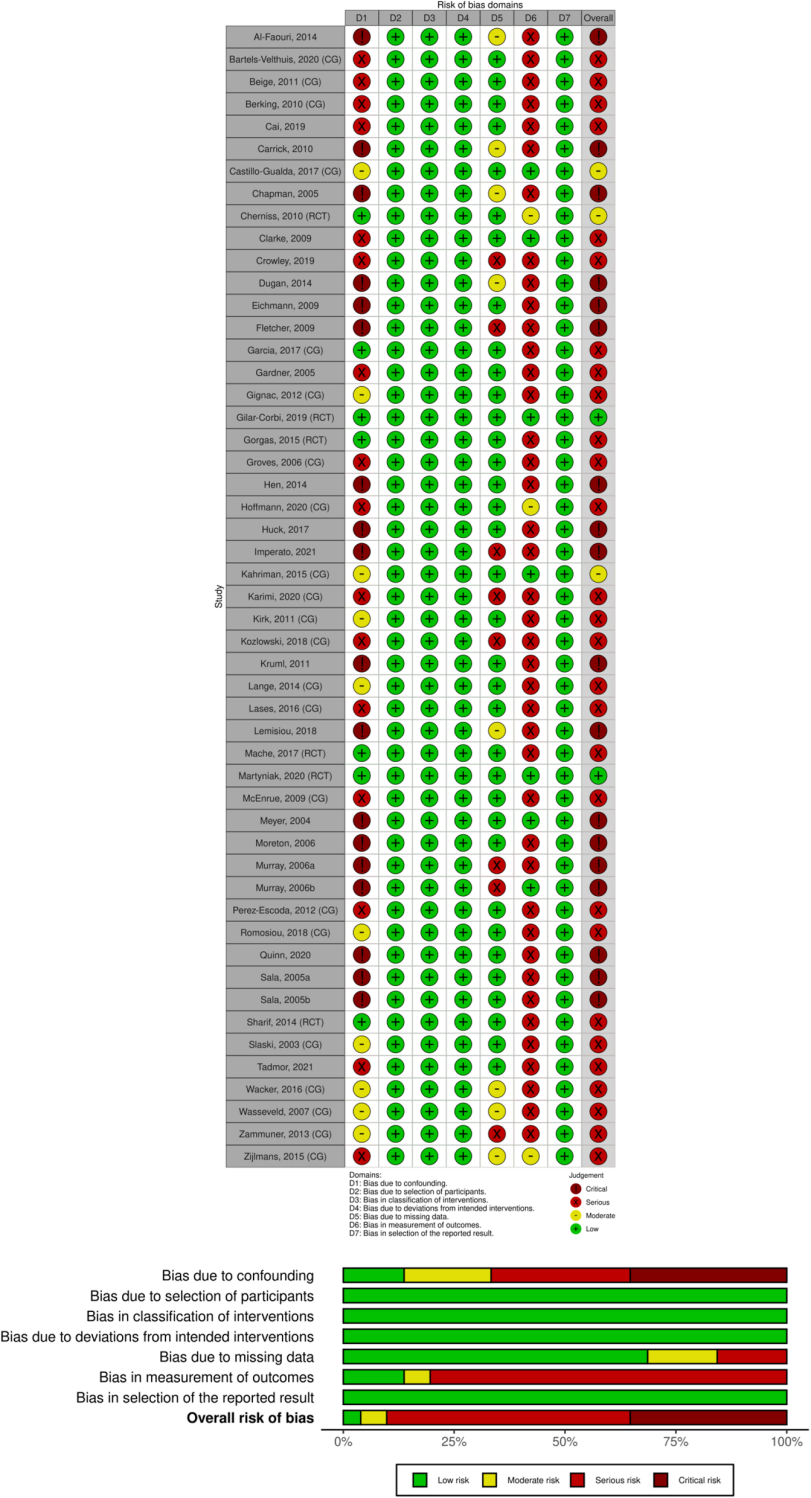
**A** Traffic Light Plot demonstrating the domain level judgements of risk of bias analysis for each study. **B** Visualized contribution of risk of bias judgements within each domain

Figure 2 visualizes the results of the risk of bias assessment with ROBINS-I. Following the assessment guidelines, there were 18 out of all 50 included studies at critical risk of bias. This overall judgement resulted from ratings of critical risk in one domain: “bias due to confounding”. All of these studies were before-after interventions with only one post-assessment. Thus, participant characteristics, like age, gender or prior knowledge, or time effects could be potential confounders. Studies were rated at high risk of bias if they did not account for potential confounders in their design or with appropriate statistical analyses. We judged 28 studies at serious risk of bias due to ratings in one or both of the following domains: “bias due to confounding” and “bias due to measurement of outcomes”. Twenty-five studies with serious risk of bias had a serious risk rating in the domain “measurement of outcomes”, because participants as well as outcome assessors were aware of the intervention. Also, the outcome measures in these studies were self- or peer-reports. Lastly, three studies were rated at overall moderate and two studies at low risk of bias. Moderate risk ratings resulted either from missing randomization or high drop-out rates. In our sensitivity analysis the exclusion of the studies with a critical risk of bias changed the effect size only marginally: *SMD*_pre-post_ = 0.40 (95% CI [0.26, 0.54], 95% PI [-0.49, 1.39], *I*^2^ = 85%, *p* < 0.01) (see Figure A, Appendix).

### Pre-post-metaanalysis

#### Main training effect

The effect size for our primary outcome, the pre-post effect sizes after four weeks, was *SMD*_pre-post_ = 0.52 (95% CI [0.32, 0.71], 95% PI [-0.57, 1.61], *I*^2^ = 91%, *p* < 0.001) (see Figure B, Appendix).

**Fig. 3:**
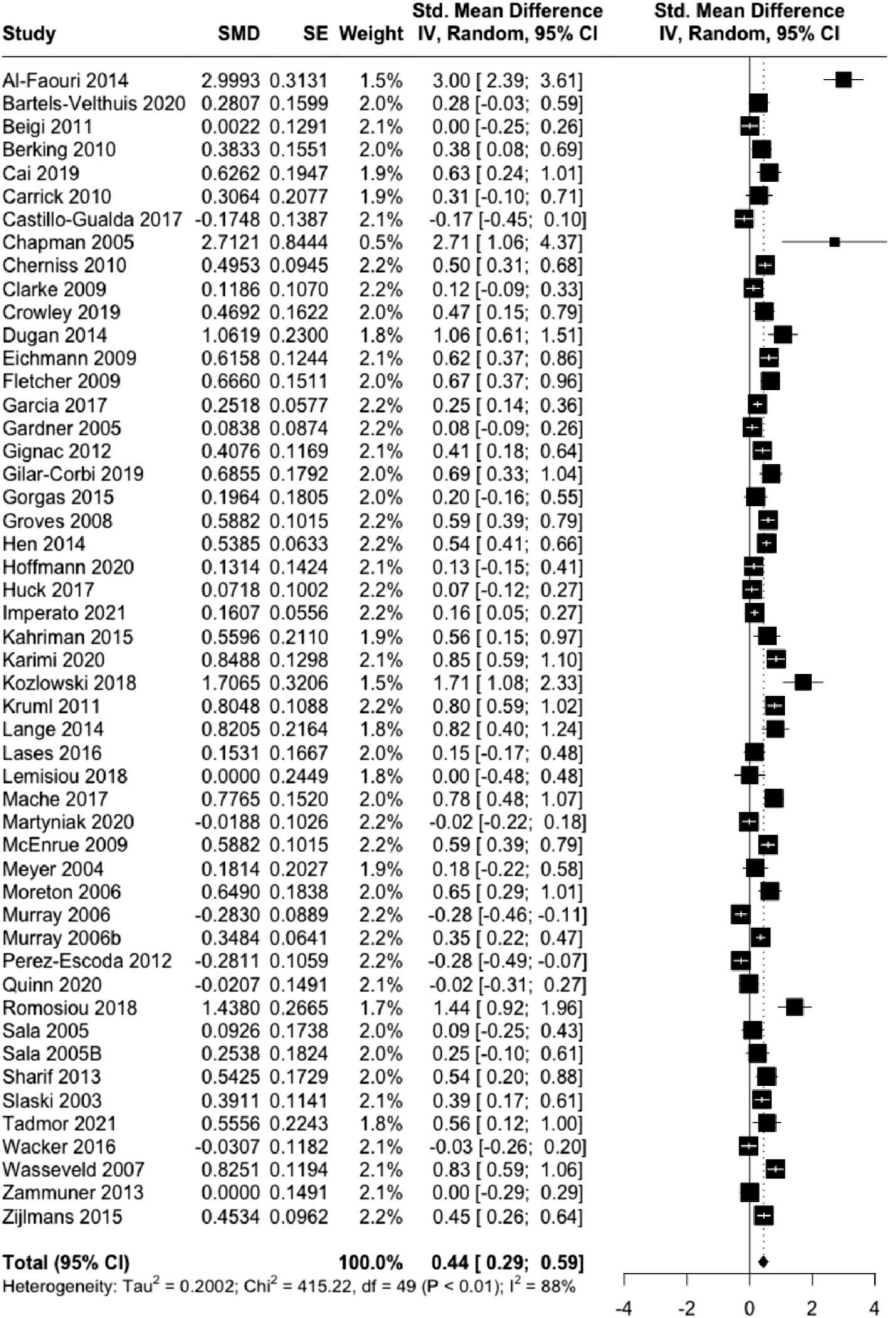
Forestplot for SMD pre-post. Effect sizes and their 95% CIs per sample for the main training effects comparing pre-to-post scores of emotional competencies are illustrated. The diamond-shaped figure shows the metanalytical computed average effect

The random-effects metaanalysis of studies with all time periods also yielded a statistically significant medium-sized effect, with *SMD*_pre-_ _post_ = 0.44 (95% CI [0.29, 0.59], 95% PI [-0.45, 1.32], *I*^2^ = 88%, *p* < 0.001). Figure 3 shows the corresponding forest plot.

Effect sizes and their 95% CIs per sample for the main training effects comparing pre-to-post scores of emotional competencies are illustrated. The diamond-shaped figure shows the metanalytical computed average effect.

The calculated funnel plot indicates a publication bias for some of the studies (see Figure C, Appendix). Egger’s regression tests for funnel plot asymmetry was significant (*t*(48) = 2.59, *p* = 0.013). Influence analysis identified one extreme outlier: Al-Faouri [83]. After omitting the outlier, a small to medium-sized effect size of *SMD*_pre-post_ = 0.38 with *I*^2^ = 86% was observed.

#### Subgroup analyses

The mixed-effect-models with all 50 studies included showed no significant difference in effect sizes between the groups for both variables – variable “construct”: *p* = 0.5; variable “profession”: *p* = 0.1. For an overview of all results, see Table 2.

**Table 2:** Summary of metaanalytic results: Main training effects and subgroup analyses

**Metaanalysis**		***k***	***SMD***	**95% *CI***	***p***	***I***^**2**^
**Treatment–Control**	Main effect	27	0.47	0.31; 0.62	< .001	68.23
Training duration	< 3 months ≥ 3 months	15 9	0.51 0.39	0.25; 0.77 0.22; 0.56	< .001 < .001	75.00 41.12
**Pre-Post**	Main effect: T1 < 4 weeks	41	0.52	0.32; 0.71	< .001	91.02
	Main effect	50	0.44	0.29; 0.59	< .001	88.03
Assessment time	Pre-Post Pre-Follow up Post-Follow up	11 11 11	0.6 0.97 0.37	0.21; 0.98 0.42; 1.52 0.01; 0.74	< .01 < .001 < .05	95.67 97.11 94.91
Construct	Emotional Intelligence Emotion Regulation Empathy Subgroup analysis	41 2 7	0.46 0.58 0.22	0.30; 0.62 0.19; 0.96 0.06; 0.39	< .001 < .001 < .001	94.49 69.49 63.86
		QM (df = 2) = 1.24,* p* = 0.53, *R*^2^ = 0.0%				
Profession	Managers Health Professionals Teachers Others Subgroup analysis	14 19 5 12	0.34 0.61 0.04 0.43	0.19; 0.49 0.32; 0.89 -0.25; 0.32 0.19; 0.66	< .001 < .001 0.7 < .001	78.14 94.67 91.66 91.60
		QM (df = 3) = 6.59, *p* = 0.08, *R*^2^ = 7.99%				

The table shows the main training effects of the pre-post- as well as treatment–control-metaanalysis and the results of our subgroup analyses investigating the impact of training duration, assessment time, EC construct and participants’ profession

#### Follow-up effect

Eleven out of the 50 studies had a follow-up assessment. The pre-post-effect of those 11 studies was medium-sized, with *SMD*_pre-post_ = 0.60 (95% CI [0.21, 0.98], *p* < 0.01, *I*^2^ = 95.67%). Comparing pre- to follow-up, we found a larger effect size of *SMD*_pre-fo_ = 0.97 (95% CI [0.42, 1.52], *p* < 0.001, *I*^2^ = 97.11%). The analysis for the mean changes between post- and follow-up measurement showed *SMD*_post-fo_ = 0.37 (95% CI [0.01, 0.74], *p* < 0.05, *I*^2^ = 94.91%).

## Comparative effect size-meta analysis

### Main training effect

**Fig. 4:**
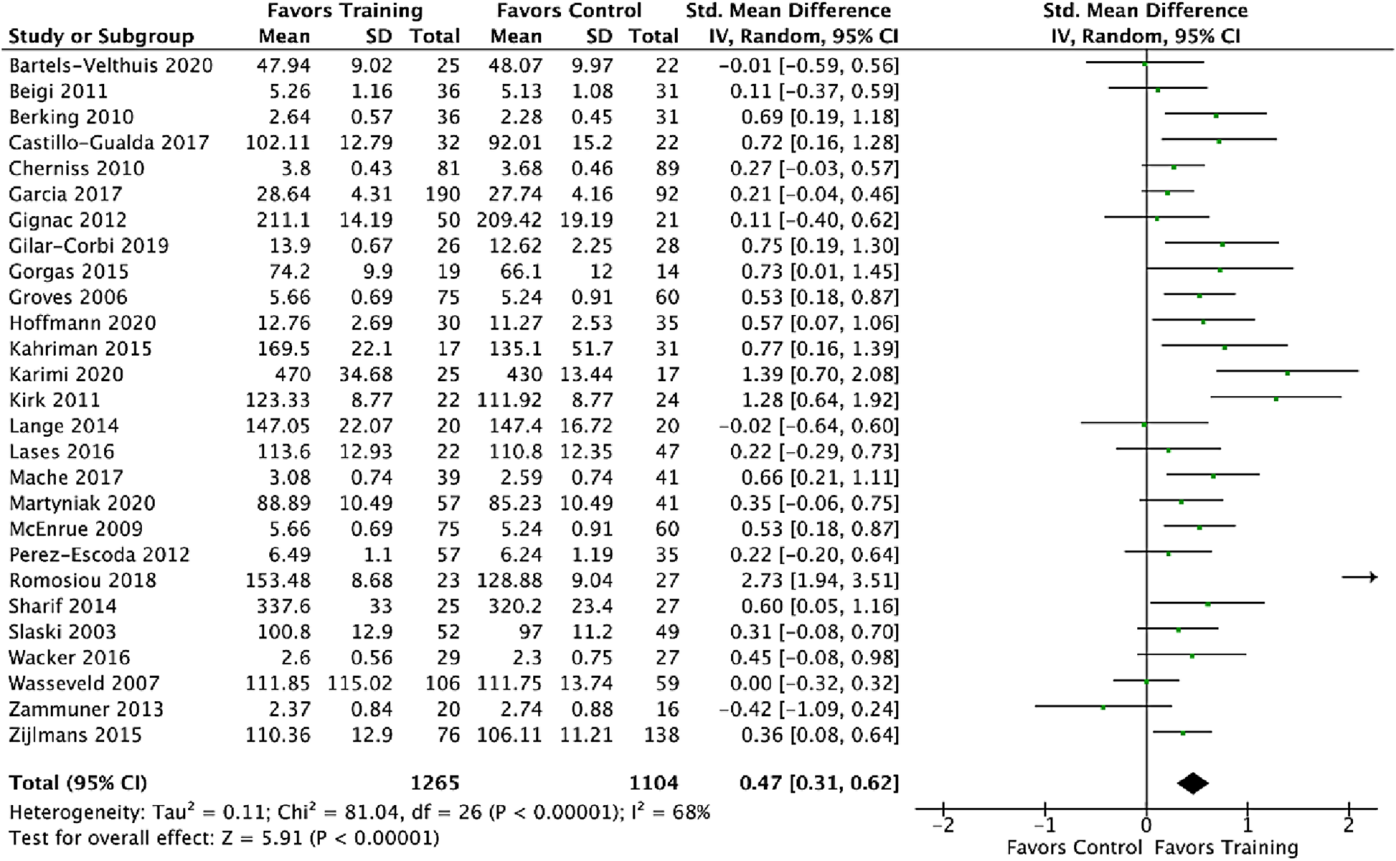
Forestplot for SMD pre-post. Post-intervention means, standard deviations, sample sizes as well as effect sizes and their 95% CIs for the main training effects comparing mean changes between intervention- and control-groups are illustrated. The diamond-shaped figure shows the metaanalytical computed average effect

The metaanalysis with *k* = 27 controlled trials showed an overall effect size of *SMD*_EG-CG_ = 0.47 (95%-CI [0.31, 0.62], 95%-PI [-0.34, 1.29], *I*^2^ = 68%). The overall training effect differs significantly from zero (*p* < 0.001). Thus, we can conclude that training had a moderate effect on emotional competencies. Figure 4 shows the corresponding forest plot.

**Fig. 5:**
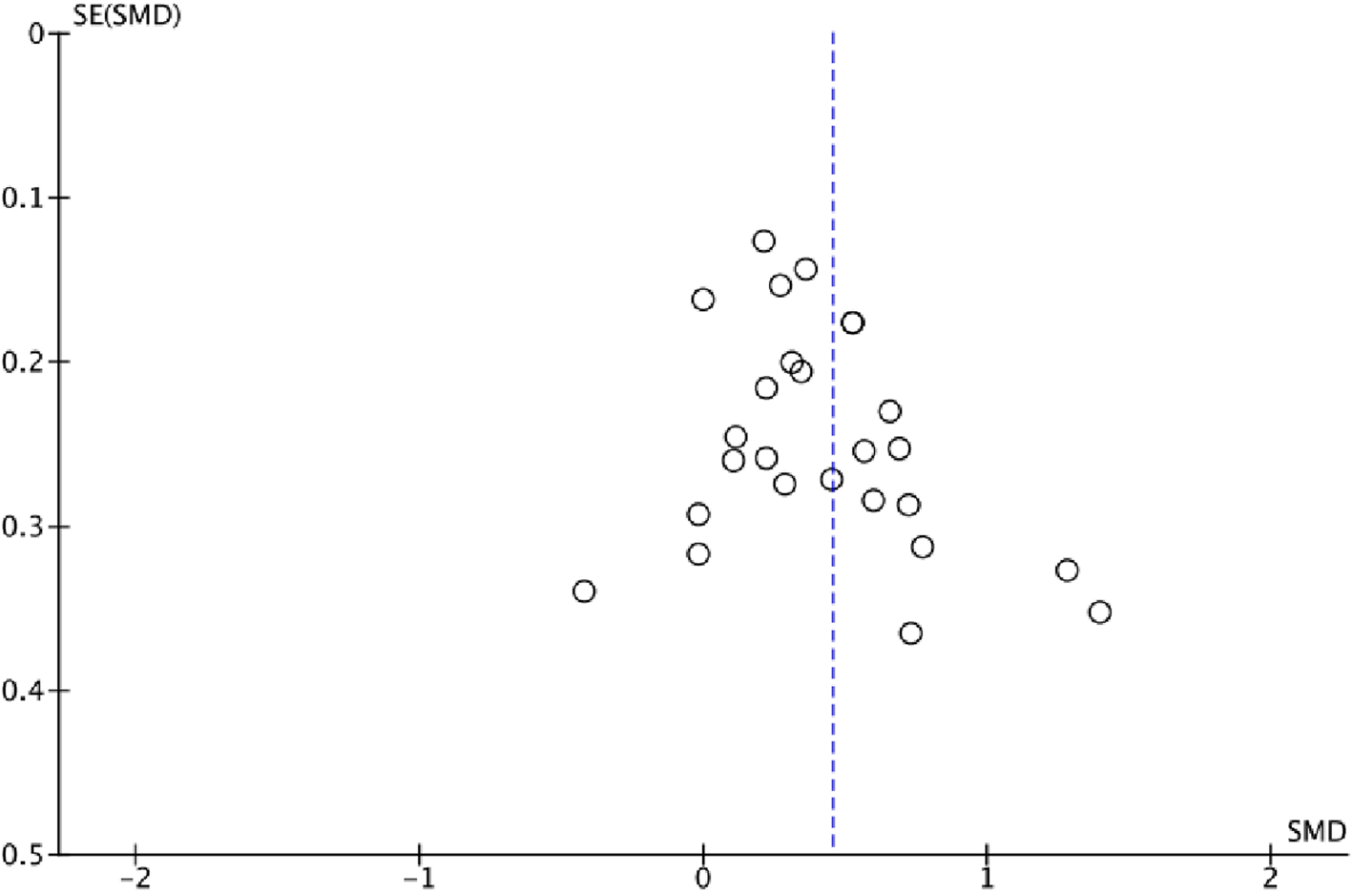
Funnel plot for the training effect of comparative effect-size metaanalysis

The funnel plot visualized in Fig. 5 indicates a publication bias. Egger’s regression tests for funnel plot asymmetry was significant (*t*(25) = 2.60, *p* = 0.02). Influence diagnostic identified one extreme outlier: Romosiou [100]. After exclusion of the outlier, a small to medium-sized effect size *SMD*_CG-EG_ = 0.40 (95% CI [0.28, 0.52], *I*^2^ = 47%) resulted.

### Effect of training duration

Fifteen interventions lasted less than three months, nine trainings had a duration of three months or more. For trainings with shorter duration we found a moderate effect size of *SMD*_EG-CG_ = 0.51 (95% CI [0.25, 0.77], *I*^2^ = 75%), after exclusion of the outlier [100] *SMD*_EG-CG_ = 0.39 (95% CI [0.22, 0.56], *I*^2^ = 41%) respectively. A small effect-size of *SMD*_EG-CG_ = 0.30 (95% CI [0.14, 0.46], *I*^2^ = 30%) resulted for the interventions with longer training duration.

## Discussion

This metaanalysis wanted to determine whether workplace interventions aiming at fostering emotional competencies are efficient and if training effects vary depending on construct and profession. We included 50 studies in our metaanalysis analyzing pre-post-effect sizes. In order to investigate training effectiveness, we included only controlled trials (*k* = 27). Both metaanalyses revealed moderate effect sizes. In response to our first research question, our results demonstrate that trainings in the working environment significantly enhance emotional competencies. This training effect persisted also three months after training end. Our findings align with previous studies investigating the training effects of emotional intelligence or empathy trainings.

For our second and forth research question, the metaanalysis showed no significant differences in training effects based on participants' professions or the specific concept of training. An explanation for our results might be the huge overlap of the included constructs regarding their theoretical models as well as training methods. In the mixed model of EI, for example, empathy and emotion regulation are facets of EI. It remains unclear, which aspects of emotional skills and abilities are actually trained and what is measured by the outcome assessors. In many EI trainings, there were many similarities with empathy trainings: for example, lectures about emotions or practicing perspective taking. So, also when trainings aim to foster different emotion-related abilities and skills, they might use similar methods and thus train the same abilities and skills. Possibly, you can always profit from a training focusing on emotional competencies, independent of the specific training focus. The question then arises: are there underlying factors that contribute to effectiveness beyond the specific objectives? It is possible, for instance, that all these trainings foster self-awareness or self-reflection, regardless of their primary goals. Additionally, as most trainings are conducted in group settings, the influence of the group—through interpersonal learning, cohesion, and feedback—has been shown to play a crucial role in facilitating intrapersonal changes [127, 128].

Also, all professions seem to profit similarly from trainings fostering emotional competencies. This is reasonable, as previous studies showed the important role of emotions and emotion-related competencies at work. Our moderator analysis only included professions with high emotional engagement. Because of this similar background, the training effects might have been similar. Furthermore, emotional competencies workshops do not only train how to consciously observe and manage emotions, but also how to interact with others. In many professions, the interaction with colleagues, supervisors or clients is daily business. So as long as participants have to deal with their emotions and needs at work and interact with others, they might profit from training independent of their profession. This suggests that regardless of the nature of the work or the specific aspect of emotional competency being targeted, these interventions yield beneficial outcomes across diverse professional contexts.

The analysis of mean change from pre- to post- and post-to-follow-up assessment showed that there is still a moderate effect from post- to follow-up assessment. This indicates that there is still some training effect more than three months after the last training day. This persistence of effect answers our third research question, suggesting that parts of the trainings had some lasting learning effect on the participants, and that they were probably able to change their behavior. About 20% of the included studies had their first follow-up assessment later than four weeks after end of intervention. The average time between the end of intervention and the first follow-up was 26 days. Some authors of the studies argued that emotional competencies need time to develop after training. In previous EI workplace interventions, changes in EI were first observed five weeks after training [129, 130]. We, therefore, decided to focus our analysis on all time points. However, our metaanalysis doesn’t support this finding. There were no significant differences in effect sizes between studies with follow-up assessment within four weeks after training and those with longer follow-up. Furthermore, the effect sizes were smaller at the second follow-up than at the first, indicating that emotional competencies don’t naturally continue to develop over time without further support. While these competencies are trainable, they may require continuous reinforcement or ongoing practice to sustain improvements. This highlights the importance of repeated interventions or follow-up training sessions to maintain or enhance the gains made from initial training.

Comparing the different trainings of emotional competencies, EI trainings seem to be the most prevalent. Depending on the branch, different emotional competence trainings are common: except for one, all empathy interventions trained health professionals, whereas EI trainings are prevalent for leadership development.

Evaluating the available information on included trainings, we can find a huge overlap in contents and methods. Most trainings fostered self-awareness through self-reflection using verbal techniques. The methods most often used were lectures, group discussions as well as role plays and other practices training EI strategies.

### Limitations

The metaanalysis is subject to limitations due to the serious and critical risk of bias present in many of the included studies. The risk of bias primarily stems from the potential for confounding variables and the subjective nature of outcome measures, but also from potential publication bias. Most of the studies didn’t have a randomized-controlled design, nor did they control for confounding variables. Thus, there is potential risk of confounding especially for the before-after-studies. The subjectivity of measures is a well-known and often discussed topic. Typically, blinding of participants and treatment providers is difficult to achieve and maintain for non-pharmacological interventions resulting in a higher risk of bias on this domain. Furthermore, the majority of studies implemented self-report measures, whereas only seven out of 50 studies used ability tests. Social desirability in self-report measure may play an even bigger role in working than in private context [131]. A study from Zammuner and colleagues [132] showed that managers’ self-ratings of their emotional competencies were higher than those of their employees. Previous studies suggest that self-report, peer-report, or ability measures assess different aspects of emotional competencies. Van Berkhout and Malouff [18], for example, showed in their metaanalysis higher effect sizes for empathy trainings using objective measures (e.g., performance tests) in comparison with trainings applying self-report measures. Looking at work-related criterion-variables, emotional competence ratings by others (e.g. employees from team leaders) correlated higher with work-related criteria like job performance or employees’ job involvement than self-report ratings [132, 133]. 360°- ratings or performance tests provide further useful information for the interpretation of training effects, especially regarding work-related criterion variables. In the evaluation of our risk assessment one must yet consider that the risk ratings were made conservatively, studies with critical risk did not influence the effect size, and that in most studies, serious or critical ratings resulted from potential risk in only one domain. While the presence of studies with critical and serious risk of bias raises concerns about the robustness of the findings, the sensitivity analysis suggests that the overall effect size estimate is relatively stable, even when excluding studies with the highest risk of bias. In sum, results must be interpreted cautiously.

A challenge we faced when conducting this metaanalysis was a lack of available data and details. Firstly, relevant data necessary for calculating effect sizes were not reported in some studies, and attempts to obtain this data through communication with authors were unsuccessful. Additionally, a majority of the included studies lacked a study protocol, hindering transparency and reproducibility. Furthermore, many study authors did not adequately describe the applied training contents, methods, and properties, making it difficult to assess the quality of the interventions and to draw conclusions about the effectiveness of specific training methods.

Statistical heterogeneity and wide prediction intervals in our analyses suggest a cautious interpretation of results. This heterogeneity may stem from missing discriminatory power of emotion-related concepts as well as differences in intervention protocols, participant characteristics, and outcome assessment methods. There was huge variability in the applied measures, especially in the assessment of EI (see Table D, Appendix). Many researches in the field of EI have already addressed concerns regarding convergent validity of different EI measures, particularly highlighting that performance-based tests and self-report measures of EI tend to correlate only weakly with each other [14, 134]. This suggests that these different types of instruments may assess distinct facets of EI, though the exact constructs each measures remain ambiguous. Given these challenges with convergent validity, we conducted a sensitivity analysis, which showed no significant differences in effect sizes between studies using performance-based assessments and those employing self-report measures (see Table E, Appendix). However, in the treatment–control metaanalysis, heterogeneity was notably lower for performance-based tests compared to self-rated measures. Thus, future studies might consider grouping by assessment tool (e.g., performance-based vs. self-report) rather than by theoretical model (e.g. ability, trait, mixed).

### Implications for practice and research

Our metaanalysis suggests that emotional competencies can be successfully trained in the working environment. Moreover, the persistence of training effects over three months after the training suggests lasting benefits, indicative of the sustainability of these programs. This is good news as many studies demonstrated the importance of emotional competencies at work. Our findings suggest that more employers and organizations should integrate emotional competence trainings as part of personnel development and health management.

There is a significant need for more high-quality, randomized studies with follow-up assessments, given the methodological limitations observed in many of the included studies, which pose a potential risk of bias. There are several ways to implement randomization also in the workplace context. Cluster-randomization, for instance, allows for the allocation of entire teams or organizations. Alternatively, recruiting participants from multiple organizations can help ensure there is no connection between training participants. In order to ensure study quality and comparability, standards for reporting studies, like the CONSORT statement [135] for randomized controlled studies, should receive more attention. Also, authors should register their studies and publish study protocols.

Additionally, authors should consider integrating more peer ratings and performance tests into their outcome assessments, as these methods offer higher criterion validity with respect to work-related criteria and greater objectivity. Given that the administration of the most commonly used ability test, the MSCEIT-2, requires over 30 min, shorter performance tests such as The Situational Test of Emotion Understanding-Brief (STEU-B) and The Situational Test of Emotion Management-B (STEM-B) [136] or the Levels of Emotional Awareness Scale (LEAS) [137] might be alternative options as they require less time.

Furthermore, additional systematic investigations exploring potential moderators of training effects would enhance our understanding of which training aspects are most effective. By comparing interventions targeting emotional competencies researchers can identify which approaches yield the most significant improvements in workplace outcomes and employee well-being. As a basis, future studies should provide comprehensive details about their training aims, contents, and methods.

A closer examination of the training content reveals a predominant focus on lectures, with limited use of practical elements, coaching or individualized feedback elements While a theoretical foundation is essential, the primary focus should ideally be on practical application and provide opportunities to reflect on situations from their daily work life. Especially in workplace settings, innovative approaches to training emotional competencies could be pursued. For instance, a more rigorous initial conceptualization phase should be emphasized. This phase would ensure that training goals are defined precisely, aligned with a solid theoretical foundation, and closely linked to targeted facets of emotional competencies, rather than broad constructs like general empathy or EI. This clarity would facilitate not only the design of targeted content and methods, but also the selection of appropriate evaluation tools, enabling a more precise assessment of training outcomes.

In practice, this targeted approach might begin with preliminary assessments of participants’ current emotional competencies, followed by individualized feedback and coaching sessions. In the next step, training programs could offer a customized pathway with specific training components that address participants’ unique needs and interest.

Additionally, most of the interventions emphasized conscious cognitive processes and the verbal expression of thoughts and emotions. Integrating non-verbal interventions—such as mindfulness-based practices, art-based activities, or body-oriented techniques—could effectively address unconscious thoughts and emotions [138]. Thus, future interventions could explore the inclusion of more-individualized training components and the use of non-verbal methods to investigate their impact on emotional competencies and determine whether these approaches yield different effect sizes compared to prior intervention studies.

First findings have linked EI with various work-related factors. However, the available data is not yet extensive enough to provide a comprehensive overview. Therefore, additional research is needed to explore the relationship between participation in emotional competence trainings and outcomes such as job performance, sick days, work stress, constructive conflict management or overall job satisfaction. This metaanalysis focused on workplace interventions designed for individuals. However, in professional settings, teams are pivotal. Numerous studies underscore the significance of team collaboration and social support [139–141]. Hence, future interventions should explore the impact of emotional competence training not only on individual performance but also on team cooperation, support, team emotional intelligence (EI), and overall team performance.

There is substantial empirical evidence that emotional competencies at work matter. For managers with personnel responsibility, poor emotional competencies are associated with low employee job satisfaction, reduced team performance, and a less cooperative team and organizational climate. Our metaanalysis demonstrates that workplace training interventions can significantly improve these competencies, underscoring the benefit of investing in employee and leadership development programs that target emotional intelligence, empathy, and emotion regulation. For sustained improvement, organizations should view emotional competency training as a long-term investment rather than a one-time initiative. Programs with regular refresher courses and advanced workshops may help participants apply their knowledge and skills in daily business and foster change processes in teams and organizations over time. Another effective approach might be regular reflective practice sessions, where workshop participants can discuss and reflect on professional interpersonal or emotional situations, allowing them to apply and enhance their emotional competencies continuously. While all professions benefit from these trainings, tailoring the programs to address the specific challenges and dynamics of different organizational cultures and professional roles could not only facilitate practical application, but also enhance participant buy-in. This customized approach may help employees see the relevance and impact of training, leading to increased engagement and productivity. For example, a healthcare institution could provide specialized empathy training for medical staff, focusing on patient care and team interaction, enhancing both patient outcomes and staff satisfaction.

By integrating these training initiatives into their organizational culture, companies can empower their workforce with essential skills for navigating the complexities of the modern workplace. Improved emotional competencies among employees and managers can lead to enhanced well-being, better communication, more effective conflict resolution, and stronger teamwork—all of which are critical for driving organizational change and success. Therefore, CEOs, health and change management professionals, as well as corporate trainers should consider integrating these trainings regularly into their employee and leadership development programs. What is necessary now is a shift in mindset. Emotional competencies should be valued and developed just as much as expertise, IQ, or management skills. By equipping employees and managers with these essential skills, CEOs can position their organizations to thrive in today's rapidly evolving business landscape, characterized by constant change and uncertainty.

## Supplementary Information

Supplementary Material 1.

## Data Availability

The data extracted and analyzed in this metaanalysis are provided in the Appendix.
