# Supplementary material for: Training emotional competencies at the workplace: a systematic review and metaanalysis
Source: BMC Psychol. 2024 Dec 4;12:718. doi: 10.1186/s40359-024-02198-3 (PMC11616120; doi:10.1186/s40359-024-02198-3)
Supplement: Supplementary file 1 — Supplementary Material 1. [file 40359_2024_2198_MOESM1_ESM.docx]

**Appendix**

**Table A**. **PICO- criteria for our metaanalysis.**

| **Population** | Healthy, working adults |
| --- | --- |
| **Intervention** | Workplace interventions training emotional competencies: EI, empathy, emotional awareness or emotion regulation |
| **Comparator** | 1. Pre- and post-intervention 2. Intervention- and control-group |
| **Outcome** | Emotional competencies (EI, empathy, emotional awareness, emotion regulation) |

**Table B**. **Search strategy EMBASE.**

1. emotional intelligence/ or emotion regulation/ or empathy/ or (emotional awareness or emotional competence* or emotional intelligence or conflict management).ab.
2. Leadership/ or manager/ or employee/ or workplace/ or occupation/ or (lead* or manage* or employ* or work* or occupation*).mp.
3. Training/ or health program/ or (training or program or intervention or workshop).ab or ((improv* adj$5 emotion*) or (improv* adj$5 empathy) or (improv* adj$5 conflict management) or (train* adj$5 conflict management) or (train* adj$5 emotion*) or (train* adj$5 empathy)).ab.
4. Intervention study/ or controlled study/or pretest-postest design/ or follow up/ or treatment outcome/ or intermethod comparison/ or compared.mp or (control* and study).mp or (control* and group*).mp.
5. 1 and 2 and 3 and 4

**Table C**. **Extracted variables with explanation.**

| Category | Variable | Explanation |
| --- | --- | --- |
|  | Authors | Names of authors |
|  | Year | Year of publication |
|  | Country | Country of data collection |
| Population characteristics | n.EG. | Sample size experimental group |
|  | n.CG. | Sample size control group |
|  | Branch | Business branch |
|  | Occupation | Type of work of participants |
|  | Job position | 0 = self-employed, 1 = leading position, 2 = employee, 3 = not specified |
|  | Gender | Percentage of females in the original sample (or EG) |
|  | Age | Average age in the original sample (or EG) |
| Training characteristics | Time pre-post | Time between pre-and post-measurement |
|  | Follow-Ups | Time between pre and follow-up measurement(s) |
|  | Number | Number of training days |
|  | Duration | Duration of training sessions in hours |
|  | Time span | Duration in total from first training day until final training day in days |
|  | Lectures | 0 = no lectures / not specified, 1 = lectures in person, 2 = live conference, 3 = videos, 4 = online, 5 = written information |
|  | Homework | Homework between workshop days  0 = no homework / not specified, 1 = practices, 2 = diary, 3 = e-learning platform |
|  | Coaching | 0 = no personal coaching / not specified, 1 = at least once personal coaching, 2 = coaching through team members |
|  | Feedback | 0 = no feedback / not specified, 1 = feedback given by group, 2 = feedback given by trainer; 3 = feedback by test results |
|  | Practices | 0 = no practices / not specified, 1 = practices training EI strategies, 2 = role play, 3 = art-based, 4 = mindfulness, 5 = games, 6 = adventure |
|  | Discussion | 0 = no discussion / not specified, 1 = live discussion, 2 = online forum discussion |
|  | Exploration | 0 = no exploration / not specified, 1 = self-directed learning, 2 = self-reflection |
|  | Modeling | 0 = no modeling / not specified, 1 = describing skills, 2 = videos, 3 = examples, 4 = trainer |
|  | Instrument | Name of instrument(s) for assessment |
|  | Reliability | Cronbach’s alpha of instrument |
|  | Correlates | Reported correlates with correlations |
|  | Dimensions | Dimensions of emotional competences trained with the intervention:  O = Overall, P = perceiving, F = facilitating thought, U = understanding, R = regulation  AS = Emotional self-awareness, AO = emotional awareness other, RS = emotional self-regulation, RO = emotional regulation other  E = empathy, C = communication, CM = conflict management |
|  | Model | For EI-trainings: model that the training is based on  0 = ability model, 1 = trait model, 2 = mixed model, 3 = not specified |
|  | Measure | Type of measure(s) used for the assessment  0 = self-report, 1 = peer-report, 2 = performance test, 3 = mixed measures, 4 = 360degree, 5 = not specified |
| Moderators | Profession | 1 = leading position, 2 = employee other than in schools and health sector, 3 = teacher, 4 = health sector, 5 = others |
|  | Construct | Concept the training is based on  0 = emotional intelligence, 1 = empathy, 2 = emotional regulation, 3 = conflict management |
| Statistical data for metaanalysis | X, SD | Reported means and standard deviations |
|  | Effect measure | Reported training effect measure, e.g. Mean difference, eta squared |
|  | p-, t-, F-value |  |
|  | CI | Confidence interval |
| Control variables | Occupation | 0 = full-time, 1 = part-time, 2 = self-employed, 3 = not specified |
|  | Gender | Percentage of females in the original sample (or EG) |
|  | Age | Average age in the original sample (or EG) |
|  | Country | Country of investigation |
|  | Year | Year of publication |
|  | CG activities | 0 = active, 1 = waiting list, 2 = passive |
|  | Randomization | 0 = no randomization, 1 = Completely randomized, 2 = quasi-randomized |
|  | Dropout | Number of people that did not take part in post-measurement in relation to pre-measurement |
|  | Blinding | Methods to blind participants |
|  | Incomplete | Completeness of reported outcome data |
|  | Publication type | PR = peer-reviewed journal, D = dissertation, C = congress paper, O = other |
|  | Bias | Risk of bias index |

**Table D1***.* **Overview of all included studies in pre-post- and treatment-control-metaanalysis. Part 1.**

| **Author(s) (Year)** | **Country** | **Publi-cation type** | **N IG** | **N CG** | **Profession** | **Follow up in months** | **RCT** | **Time span** | **t1** | **Training days** | **Duration in h** | **Training focus** | **EC measures** | **Other outcomes** |
| --- | --- | --- | --- | --- | --- | --- | --- | --- | --- | --- | --- | --- | --- | --- |
| Al-Faouri et al. (2014) [83] | JOR | PR | 70 |  | Nurses |  |  | 7 weeks | te | 7 | 14 | EI | GENOS | JSS |
| Bartels-Velthuis et al. (2020) [84] | NED | PR | 25 | 22 | Health care professionals |  |  | 9 weeks | te | 9 |  | E | EQ | FFMQ SCS PSS ProQoL |
| Beigi & Shiromohammadi (2011) [85] | IR | PR | 56 | 150 | Bankers |  |  | 2 months | 2 weeks | 8 |  | EI | ECI-2 | Service quality rating |
| Berking, Meier & Wupperman (2010) [86] | CH | PR | 36 | 31 | Police officers |  |  | 6 weeks | te | 3 | 9 | ER | ERSQ PANAS |  |
| Cai et al. (2019) [87] | US | PR | 22 |  | Physicians | 1, 3, 6 |  |  | te |  |  | E | JSPE |  |
| Carrick (2010) [88] | US | D | 15 |  | Nurse manager |  |  | 1 week | 4 months | 2 | 5 | EI | EQ-i |  |
| Castillo-Gualda et al. (2017) [89] | ES | PR | 32 | 22 | Teacher |  |  | 3 months | 9 months | 8 | 24 | EI | MSCEIT | UWES MBI |
| Chapman (2005) [90] | AUS | B | 150 |  | Head of Housing Services |  |  |  |  | 5 |  | EI | BEIQ EIQ:M 360 |  |
| Cherniss, Grimm & Liautaud (2010) [60] | US | PR | 81 | 89 | Manager | 24 | yes | 2 years | 1 year after ts |  |  | EI | ECI 360 |  |
| Clarke (2010) [53] | UK | PR | 53 | 18 | Project manager | 6 |  | 2 days | 2 months | 2 |  | EI | MSCEIT |  |
| Crowley et al. (2019) [91] | US | PR | 27 |  | Veterinary professionals |  |  | 5 days | 1 year |  |  | EI | TEIQue-SF | AAQ-II RSA Communication skills |
| Dugan et al. (2014) [92] | US | PR | 22 |  | Physicians | 12, 24 |  | 7 years | 1 year after ts |  |  | EI | EQ-i | Patient satisfaction |
| **Author(s) (Year)** | **Country** | **Publi-cation type** | **IG** | **CG** | **Profession** | **Follow up** | **RCT** | **Time span** | **t1** | **Training days** | **Duration** | **Training focus** | **EC measures** | **Other outcomes** |
| Fletcher et al. (2009) [93] | UK | PR | 50 |  | Physicians |  |  | 7 months | 2 weeks |  |  | EI | EQ-i |  |
| Garcia et al. (2017) [94] | ES | PR | 190 | 92 | Work team supervisors | 3 |  | 3 days | 3 months | 3 | 16 | E | TMMS-24  TECA |  |
| Gardner (2005) [95] | AUS | D | 79 |  | Teacher | 1 |  | 5 weeks | te | 5 | 10 | EI | SUEIT | JSS; OCS; ORQ: Health Neuroticism |
| Gignac et al. (2012) [74] | AUS | PR | 50 | 21 | Pharmaceutical sales representatives |  |  | 3 months | te | 5 | 10,5 | EI | GENOS-self & rater-report |  |
| Gilar-Corbi et al. (2019) [96] | ES | PR | 26 | 28 | Senior manager | 12 | yes | 7 weeks | te | 7 | 30 | EI | EQ-i STEU STEM |  |
| Gorgas et al. (2015) [54] | US | PR | 19 | 14 | EM residents | 6 | yes | 1 day | te | 1 |  | EI | ECI 360 |  |
| Groves, McEnrue & Shen (2008) [97] | US | PR | 75 | 60 | Employees |  |  | 11 weeks | te |  |  | EI | EISDI |  |
| Hen & Sharabi-Nov (2014) [98] | ISR | PR | 186 |  | Teacher |  |  | 14 weeks | te |  | 56 | EI | SSREIT | IRI Reflective diary |
| Hoffmann, Ivcevic & Maliakkal (2020) [99] | ES | PR | 30 | 36 | Professional adults | 2 |  | 4 weeks | te | 8 | 8 | EI | STEU-B |  |
| Huck (2017) [100] | US | D | 60 |  | HR Manager |  |  | 3 weeks | te | 2 | 8 | EI | GENOS 360 |  |
| Imperato & Paul (2021) [101] | US | PR | 198 |  | Phyisicans |  |  | 1 year | te |  |  | EI, E | WLEIS | JSE |
| Kahriman et al. (2016) [102] | TR | PR | 17 | 31 | Nurses |  |  | 4 months | te | 5 | 20 | E | ESS |  |
| Kirk et al. (2011) [103] | AUS | PR | 24 | 22 | Employees |  |  | 3 days | 2 weeks | * | * | EI | SEIS | PANAS ESES |
| Kozlowski et al. (2018) [70] | AUS | PR | 30 | 30 | Nurses |  |  | 4 weeks | 3 months | 1 | 4 | EI | GENOS |  |
| **Author(s) (Year)** | **Country** | **Publi-cation** | **IG** | **CG** | **Profession** | **Follow up** | **RCT** | **Time span** | **t1** | **Training days** | **Duration** | **Training focus** | **EC measures** | **Other outcomes** |
| Kruml & Yockey (2011) [104] | US | PR | 78 |  | Employees |  |  |  | te |  | 37 | EI | EQ-i |  |
| Lange (2014) [105] | NA | D | 20 | 20 | Judicial employee | 3 |  | 1 week | te | 4 |  | EI | TeiQue-SF |  |
| Lases et al. (2016) [106] | NED | PR | 22 | 47 | Surgical residents |  |  | 3 months | te | 5 |  | E | JSPE |  |
| Lemisiou (2018)[107] | GR | PR | 10 |  | Manager |  |  | 1 year | te |  |  | EI | ESCI 360 |  |
| Mache et al. (2017)[108] | GER | PR | 40 | 40 | Physicians | 4 & 9 | yes | 3 months | te | 12 | 18 | ER | ERSQ | PSQ COPSOQ MBI JSS |
| Martyniak & Pellitteri (2020) [109] | PL | PR | 60 | 44 | Teacher |  | yes | 3 months | te | 3 |  | EI | MSCEIT |  |
| McEnrue, Groves & Shen (2009) [110] | US | PR | 75 | 60 | Employees |  |  | 3 months | te |  |  | EI | EISDI |  |
| Meyer, Fletcher & Parker (2004)[111] | US | PR | 15 |  | Dentist |  |  | 1 day | te | 1 |  | EI | MSCEIT |  |
| Moreton (2018) [112] | US | D | 24 |  | Manager |  |  | 6 months | te |  |  | EI | EQ-i |  |
| Murray et al. (2006a) [113] | AUS | C | 108 | 327 | Employees |  |  | 2,5 weeks | te | 2 | 12 | EI | WEIp-66 | Observed task performance |
| Murray et al. (2006b) [113] | AUS | C | 264 |  | Employees |  |  | 2,5 weeks | te | 3 | 20 | EI | WEIP-6 | Task performance Observed performance |
| Quinn et al. (2020) [114] | US | PR | 27 |  | Physician, resident |  |  | 6 months | te | 9 |  | E | IRI | ESI MBI |
| Romosiou, Brouzos & Vassilopulos (2019) [115] | GR | PR | 23 | 27 | Police officers | 3, 24 |  | 5 weeks | te | 4 |  | EI | SEIS | IRI PSS CD-RISC |
| **Author(s) (Year)** | **Country** | **Publi-cation** | **IG** | **CG** | **Profession** | **Follow up** | **RCT** | **Time span** | **t1** | **Training days** | **Duration** | **Training focus** | **EC measures** | **Other outcomes** |
| Sala (2002a) [116] | BR | PR | 20 |  | Manager |  |  | 1 year | te | 5 |  | EI | ECI |  |
| Sala (2002b) [116] | BR | PR | 19 |  | Accountants |  |  | 1 year | 2 months |  |  | EI | ECI |  |
| Sharif & Keshavarzi (2013) [117] | IR | PR | 25 | 27 | Nurse | 1 | yes | 2 days | te | 2 | 16 | EI | EQ-i GHQ-20 |  |
| Slaski (2003) [118] | GB | PR | 60 | 60 | Manager |  |  | 4 weeks | 6 months | 4 |  | EI | EQ-i | EQI GHQ-28 |
| Tadmor et al. (2016) [119] | ISR | PR | 16 | 15 | Medical staff |  |  | 5 months | te | 10 | 20 | EI | EQ-i |  |
| Wacker & Dziobek (2018) [120] | GER | PR | 43 | 61 | Medical staff |  |  | 3 days | 3 months | 3 | 21 | E | SPF | stressors at work  NVC |
| Wasseveld, Overbeeke & Derksen (2007) [121] | NED | PR | 123 | 59 | Manager |  |  | 6 months | te | 6 |  | EI | EQ-i |  |
| Zammuner et al. (2013) [73] | IT | PR | 27 | 22 | Manager |  |  | 7-10 days | 1 month |  |  | EI | ECI - self and other | Life satisfaction  JIS |
| Zijlmans et al. (2015) [122] | NED | PR | 76 | 138 | Nurses | 3 |  | 2 days | te | 2 |  | EI | EQ-i | ERCBS-K  CISS-21 |
| N CG = sample size control group, N IG = sample size intervention group, RCT = randomized controlled trial, EC measures = emotional competencies measures; PR = Peer-reviewed journal, D = dissertation; C = conference paper. Ts = training start, te = training end. EI = emotional intelligence, E = empathy, ER = Emotion regulation. SR = self-report, PR = peer report or 360°-feedback, PT = performance test. EQ = Empathy Quotient , ECI = Emotional Competence Inventory , ERSQ = Emotion Regulation Skills Questionnaire, PANAS = The Positive and Negative Affect Scale, JSPE = Jefferson Scale of Empathy, EQ-I = Emotional Quotient Inventory, TEIQue = Trait Emotional Intelligence Questionnaire, TMMS = Trait Meta Mood Scale, TECA = Cognitive and Affective Empathy, SUEIT = Swinburne University Emotional Intelligence Test, STEU = The Situational Test of Emotional Understanding, STEM = The Situational Test of Emotion Management, WLEIS = Wong and Law Emotional Intelligence Scale, ESS = Empathic Skill Scale, EISDI = Emotional Intelligence Self-Description Inventory, MSCEIT = Mayer-Salovey-Caruso Emotional Intelligence Test, BEIQ = Boston Emotional Intelligence Questionnaire, WEIP-6 = Work Group Emotional Intelligence Profile, GROP = Emotional Development Inventory for Adults, IRI = Interpersonal Reactivity Index, SEIS = Schutte Emotional Intelligence Scale, SPF = Saarbrücker Persönlichkeitsfragebogen. JSS = Job Satisfaction Survey, FFMQ = Five Facet Mindfulness Questionnaire, SCS = Self-compassion Scale, OCS = Organizational Commitment Scale, ORQ = Occupational Roles Questionnaire, PSS = Perceived Stress Scale, UWES = Utrecht Work Engagement Scale, MBI = Maslach Burnout Inventory, AAQ-II = Acceptance and Action Questionnaire, RSA = Resilience Scale for Adults, GWB = General Well-Being Questionnaire Scale, PES = Psychological Empowerment Scale, ESE = The Emotional Self-Efficacy, Scale, PSQ =Perceived Stress Questionnaire, COPSOQ = Copenhagen Psychosocial Questionnaire, ESI = Emotional Styles Inventory, GHQ = General Health Questionnaire, NVC = Nonviolent Communication, JIS = Job Involvement Scale, ERCBS-K = Emotional Reactions to Challenging Behaviour Scale, CISS-21 = Coping Inventory for Stressful Situations.  *self-administered training | | | | | | | | | | | | | | |

**Table D2***.* **Overview of all included studies in pre-post- and treatment-control-metaanalysis. Part 2.**

| **First Author (Year)** | **Post hoc exlusion** | **Mean Pre** | **SD Pre** | **Mean Post IG** | **SD Post** | **Mean Post CG** | **SD Post CG** | **Home-work** | **Coach-ing** | **Feed-back** | **Pract-ices** | **Discuss-ion** | **Explor-ation** | **Model-ing** | **Lectu-res** |
| --- | --- | --- | --- | --- | --- | --- | --- | --- | --- | --- | --- | --- | --- | --- | --- |
| Al-Faouri (2014) |  | 81,94 | 9,71 | 111,5 | 9,15 |  |  | 0 | 0 | 0 | 1,2 | 1 | 0 | 0 | 1 |
| Bartels-Velthuis (2020) | | 45,47 | 8,52 | 47,94 | 9,02 | 48,07 | 9,97 | 1 | 0 | 0 | 1,2,4 | 0 | 2 | 0 | 0 |
| Beigi (2011) |  | 3,8 | 31 | 3,87 | 0,38 | 5,13 | 1,08 | 0 | 0 | 3 | 0 | 1 | 0 | 3 | 1 |
| Berking (2010) |  | 2,47 | 0,71 | 2,75 | 0,6 | 2,28 | 0,45 | 1 | 0 | 0 | 1,4 | 0 | 1,2 | 0 | 1 |
| Cai-Fei (2019) |  | 113,1 | 10,6 | 120 | 9,8 |  |  | 0 | 0 | 1 | 2 | 1 | 0 | 3 | 1 |
| Carrick (2010) |  | 104,55 | 9,81 | 107,73 | 8,65 |  |  | 0 | 1 | 3 | 0 | 0 | 0 | 3 | 1 |
| Castillo-Gualda (2017) |  | 102,61 | 2,79 | 102,11 | 12,79 | 92,01 | 4,16 | 0 | 0 | 0 | 1 | 1 | 0 | 0 | 1 |
| Castillo-Gualda (2019) | x |  |  |  |  |  |  | 0 | 0 | 0 | 1 | 1 | 2 | 0 | 1 |
| Chapman (2005) |  | 37 | 2,48 | 45 | 1,72 |  |  | 2 | 1 | 0 | 0 | 0 | 0 | 0 | 1 |
| Cherniss (2010) |  | 3,705 | 0,39 | 3,9 | 0,39 | 3,68 | 0,46 | 1 | 1 | 1 | 0 | 1 | 2 | 4 | 0 |
| Clarke (2009) |  | 95,8 | 14,71 | 97,57 | 16,12 |  |  | 0 | 0 | 0 | 0 | 0 | 0 | 0 | 1 |
| Crowley (2019) |  | 149,8 | 18 | 158,5 | 16 |  |  | 0 | 0 | 1 | 1, 2 | 1 | 2 | 0 | 1 |
| Dugan (2014) |  | 104,74 | 6,09 | 111,45 | 4,92 |  |  | 1 | 0 | 3 | 2 | 1 | 0 | 3 | 1 |
| Edelman (2016) | x | 98,88 | 12,46 | 106,67 | 11,39 |  |  | 0 | 0 | 0 | 1 | 0 | 0 | 1 | 1 |
| Eichmann (2009) |  | 95,9 | 11,9 | 104 | 10,1 |  |  | 1 | 1 | 3 | 0 | 1, 2 | 2 | 0 | 1 |
| Fletcher (2009) |  | 95,9 | 11,9 | 104 | 10,1 |  |  | 0 | 0 | 0 | 0 | 0 | 0 | 0 | 0 |
| Garavan (2015) | x |  |  |  |  |  |  | 0 | 0 | 0 | 3 | 1 | 2 | 0 | 0 |
| Garcia (2017) |  | 27,51 | 4,47 | 28,64 | 4,31 | 27,74 | 4,16 | 0 | 0 | 0 | 1 | 0 | 2 | 0 | 1 |
| Gardner (2005) |  | 178,1 | 18,9 | 179,7 | 20,23 |  |  | 1, 2 | 0 | 0 | 1, 2 | 1 | 2 | 3 | 1 |
| Gignac (2012) |  | 204,84 | 15,12 | 211,1 | 14,19 | 209,42 | 19,19 | 0 | 1 | 2 | 2 | 0 | 0 | 0 | 1 |
| Gilar-Corbi (2019) |  | 12,38 | 2,15 | 13,9 | 0,67 | 12,62 | 2,25 | 3 | 0 | 0 | 1 | 1,2 | 1 | 3 | 1,3 |
| Gorgas (2015) |  | 62,6 | 11,7 | 65 | 11,7 | 66,1 | 12 | 0 | 0 | 0 | 0 | 1 | 0 | 2 | 1 |
| Grissom (1986) | x |  |  |  |  |  |  |  |  |  |  |  |  |  |  |
| Groves (2006) |  | 5,25 | 0,69 | 5,66 | 0,69 | 5,24 | 0,91 | 1,2 | 2 | 3 | 2 | 1 | 2 | 2 | 1 |
| Hen (2014) |  | 129,16 | 5,4 | 132,08 | 14,32 |  |  | 2 | 0 | 0 | 1,2 | 1 | 2 | 1 | 1 |
| Hoffmann (2020) |  | 12,33 | 2,52 | 12,67 | 2,69 | 11,27 | 2,53 | 0 | 1 | 1 | 2, 3 | 1 | 2 |  | 1 |
| Huck (2017) |  | 3,91 | 0,55 | 3,95 | 0,5 |  |  | 0 | 0 | 0 | 0 | 0 | 0 | 0 | 1 |
| **First Author** | **Post hoc exlusion** | **Mean Pre** | **SD Pre** | **Mean Post IG** | **SD Post** | **Mean Post CG** | **SD Post CG** | **Home-work** | **Coach-ing** | **Feed-back** | **Pract-ices** | **Discuss-ion** | **Explor-ation** | **Model-ing** | **Lectu-res** |
| Hülsheger (2014) | x |  |  |  |  |  |  |  |  |  |  |  |  |  |  |
| Imperato (2021) |  | 5,4 | 0,62 | 5,5 | 0,81 |  |  | 0 | 0 | 1 | 0 | 1 | 0 | 0 | 0 |
| Kahriman (2015) |  | 146,7 | 38,8 | 169,5 | 22,1 | 135,1 | 51,7 | 0 | 0 | 1 | 1,2,3 | 1 | 2 | 0 | 1 |
| Karimi (2020) |  | 434 | 41,8434 | 470 | 41,8434 | 430 | 13,44 | 1 | 0 | 0 | 4 | 1 | 2 | 0 | 1 |
| Kirk (2011) | x | 3,77 | 0,18 | 4,09 | 0,25 | 111,92 | 8,77 | 2 | 0 | 0 | 0 | 0 | 0 | 0 | 0 |
| Kozlowski (2018) |  |  |  |  |  |  |  | 1 | 0 | 1 | 1 | 0 | 0 | 0 | 1 |
| Kruml (2011) |  | 99,69 | 5,8078 | 104,41 | 5,8078 |  |  |  |  | 3 | 2 |  | 2 | 3 | 1 |
| Lange (2014) |  | 133,1 | 16,32 | 147,05 | 22,07 | 147,4 | 16,72 | 0 | 0 | 0 | 1 | 1 | 0 | 3 | 1 |
| Lases (2016) |  | 111,77 | 11,52 | 113,6 | 12,93 | 110,8 | 12,35 | 0 | 0 | 0 | 4 | 0 | 2 | 3 | 1 |
| Lemisiou (2018) |  | 3,67 | 0,355 | 3,67 | 0,237 |  |  | 0 | 1 | 3 | 0 | 0 | 0 | 0 | 0 |
| Mache (2017) |  | 2,47 | 0,77 | 3,08 | 0,74 | 2,59 | 0,74 | 1 | 0 | 0 | 1,4 | 1 | 0 | 2 | 1 |
| Martyniak (2020) |  | 86,42 | 9,46 | 86,24 | 5,6577 | 85,23 | 10,49 | 1 | 0 | 0 | 1 | 0 | 0 | 0 | 1 |
| McEnrue (2009) |  | 5,25 | 0,69 | 5,66 | 0,69 | 5,24 | 0,91 | 2 | 0 | 1,2 | 1 | 0 | 2 | 0 | 1 |
| Meyer (2004) |  | 107,89 | 10,89 | 109,98 | 10,94 |  |  | 0 | 0 | 0 | 6 | 1 | 0 | 0 | 0 |
| Moreton (2006) |  | 96,1 | 14,9 | 106,1 | 15 |  |  | 1 | 1 | 2 | 1 | 0 | 0 | 0 | 0 |
| Murray (2006a) |  | 4,88 | 0,14 | 4,84 | 0,14 |  |  |  |  |  |  |  |  |  |  |
| Murray (2006b) |  | 4,86 | 0,2 | 4,93 | 0,2 |  |  |  |  |  |  |  |  |  |  |
| Nicotera (2014) | x |  |  |  |  |  |  | 0 | 0 | 0 | 1 | 1 | 2 | 3 | 1 |
| Perez-Escoda (2012) |  | 133,1 | 16,32 | 128,45 | 26,34 | 6,24 | 1,19 | 0 | 0 | 0 | 1,2,4 | 1 | 2 | 0 | 1 |
| Quinn (2020) |  | 17,5 | 6,1 | 17,37 | 6,7 |  |  | 0 | 0 | 0 | 1,2,3,4 | 0 | 0 | 0 | 1 |
| Roger (1995) | x |  |  |  |  |  |  |  |  |  |  |  |  |  |  |
| Romosiou (2018) |  | 135,13 | 12,32 | 153,48 | 8,68 | 128,88 | 9,04 | 1 | 0 | 0 | 1,2 | 1 | 2 | 0 | 1 |
| Sala (2005) |  | 3,47 | 1,14 | 3,58 | 0,76 |  |  |  |  |  |  |  |  |  |  |
| Sharif (2014) |  | 3,77 | 0,83 | 3,99 | 0,82 | 320,2 | 23,4 | 0 | 0 | 0 | 1 | 1 | 0 | 0 | 1 |
| Slaski (2003) |  | 319 | 33,2 | 337,6 | 33 | 97 | 11,2 | 2 | 0 | 3 | 1, 2 | 1 | 0 | 0 | 1 |
| Tadmor (2021) |  | 95,6 | 13,1 | 100,8 | 12,9 |  |  | 0 | 0 | 3 | 1,2 | 1 | 2 | 2 | 1 |
| Turner (2008) | x |  |  |  |  |  |  | 1 | 0 | 0 | 0 | 1 | 0 | 2 | 1 |
| Wacker (2016) |  | 97,9 | 13,1 | 105,6 | 12,9 | 2,3 | 0,75 | 0 | 0 | 0 | 1,2 | 1 | 2 | 0 | 1 |
| Wagstaff (2012) | x |  |  |  |  |  |  | 2 | -1 | 0 | 1,2 | 1 | 2 | 2,3 | 1 |
| **First Author** | **Post hoc exlusion** | **Mean Pre** | **SD Pre** | **Mean Post IG** | **SD Post** | **Mean Post CG** | **SD Post CG** | **Home-work** | **Coach-ing** | **Feed-back** | **Pract-ices** | **Discuss-ion** | **Explor-ation** | **Model-ing** | **Lectu-res** |
| Wasseveld (2007) |  | 2,62 | 0,64 | 2,6 | 0,56 | 111,75 | 13,74 | 0 | 0 | 3 | 1 | 1 | 0 | 0 | 1 |
| Zammuner (2013) |  | 100,03 | 14,16 | 111,85 | 15,02 | 2,74 | 0,88 | 3 | 0 | 0 | 0 | 0 | 2 | 0 | 4 |
| Zijlmans (2015) |  | 2,59 | 0,87 | 2,59 | 0,75 | 106,11 | 11,21 | 0 | 0 | 2,3 | 1 | 0 | 0 | 0 | 1 |
| Zijlmans (2011) | x | 104,36 | 13,1 | 110,36 | 12,9 |  |  | 1 | 1 | 2,3 | 1,2 | 1 | 0 | 0 | 1 |
| For explanations of the extracted variables, see Table C in the Appendix. | | | | | | | | | | | | | | | |

| **Metaanalysis** |  | ***k*** | ***SMD*** | **95% *CI*** | ***p*** | ***I*^2^** |
| --- | --- | --- | --- | --- | --- | --- |
| Treatment- Control | Performance tests  Self-rated tests  Subgroup analysis | 4  23 | 0.55  0.45 | 0.30; 0.80  0.28; 0.63 | < .001  < .001 | 0.00  72.00 |
|  |  | QM (df = 1) = 0.39, *p* = 0.53 | | | | |
| Pre-Post | Performance tests  Self-rated tests  Subgroup analysis | 6  44 | 0.13  0.48 | -0.08; 0.35  0.34; 0.63 | < .001  < .001 | 73.66  93.88 |
|  |  | QM (df = 1) = 2.69, *p* = 0.10, *R*^2^ = 3.85 % | | | | |

**Table E***.* **Subgroup analysis of treatment-control and pre-post studies: Performance tests versus self-rated measures.**

**Fig A**. **Forestplot for SMD pre-post after exclusion of studies with “critical” risk of bias rating in ROBINS-I.**


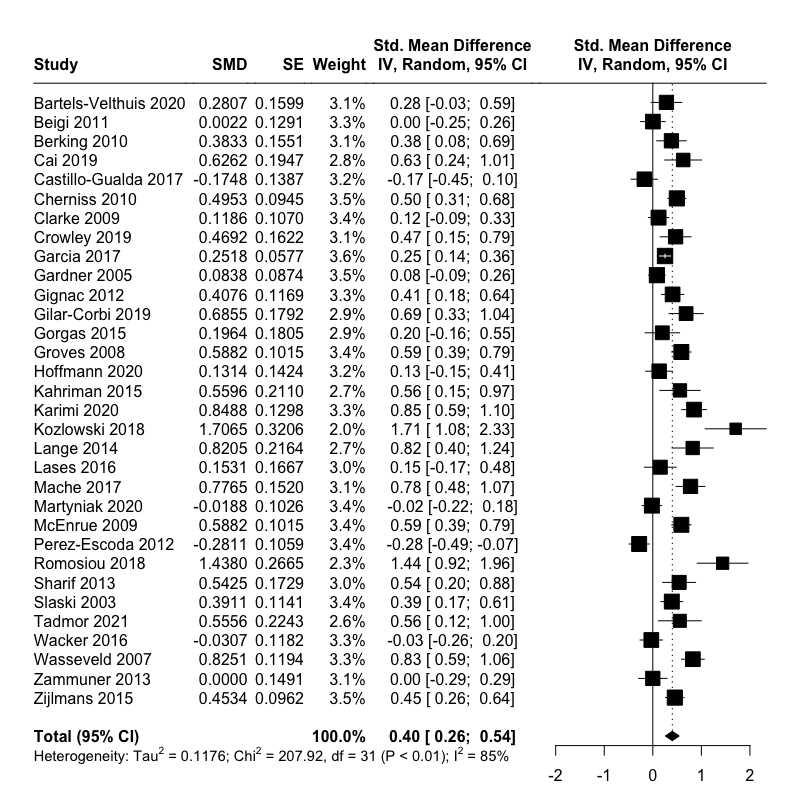


**Fig B. Forestplot for SMD pre-post of emotional competencies assessed within four weeks after training end.**
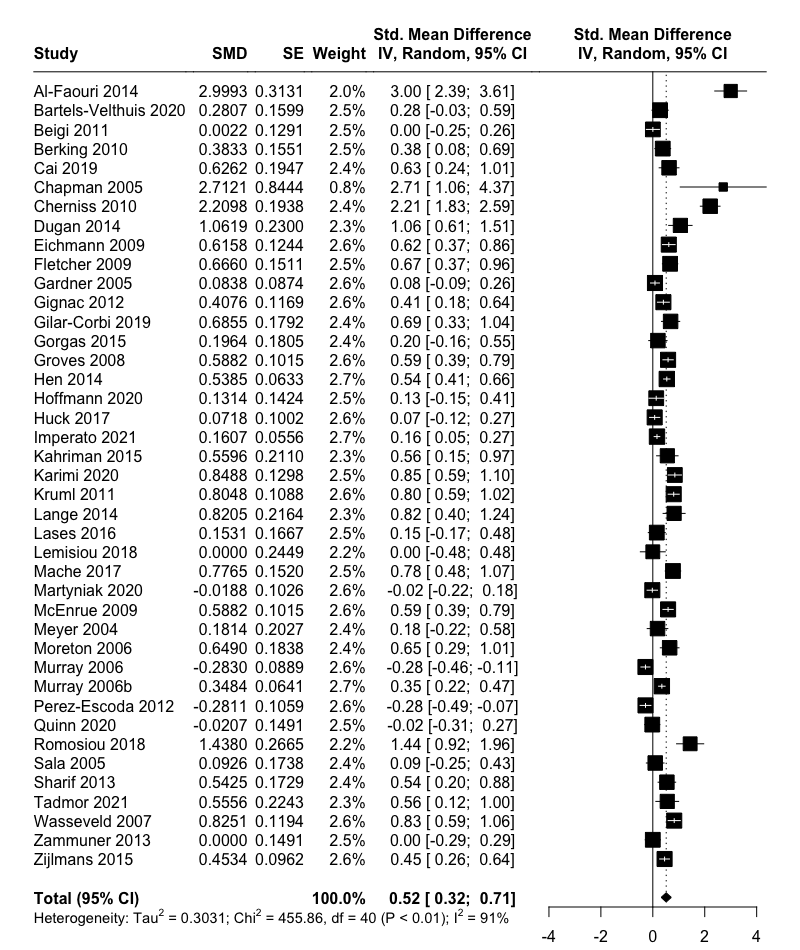


**Figure C. Funnelplot for the main training effect pre-post.**


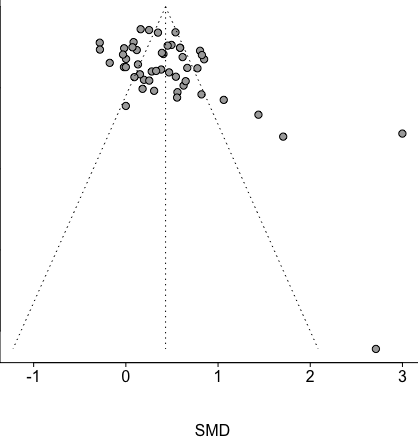


Effect sizes for the emotional competence change comparing the scores between pre- and post- assessment are plotted as observed outcomes against standard errors.
